# Using Deep Learning Neural Networks to Improve Dementia Detection: Automating Coding of the Clock-Drawing Test

**DOI:** 10.21203/rs.3.rs-4909790/v1

**Published:** 2024-10-15

**Authors:** Mengyao Hu, Tian Qin, Richard Gonzalez, Vicki Freedman, Laura Zahodne, Edmundo Melipillan, Yi Murphey

**Affiliations:** The University of Texas Health Science Center at Houston; University of Michigan–Dearborn; University of Michigan–Ann Arbor; University of Michigan–Ann Arbor; University of Michigan–Ann Arbor; University of Michigan–Ann Arbor; University of Michigan–Dearborn

## Abstract

Alzheimer’s disease and related dementias (ADRD) is a growing public health concern. The clock-drawing test (CDT), where subjects draw a clock, typically with hands showing 11:10, has been widely used for ADRD-screening. A limitation of including CDT in large-scale studies is that the CDT requires manual coding, which could result in biases if coders interpret and implement coding rules differently. This study created and evaluated an intelligent CDT Clock Scoring system built with Deep Learning Neural Networks (DLNN) to automatically code CDT images. We used a large, publicly available repository of CDT images from the 2011–2019 National Health and Aging Trends Study (NHATS) and compared three advanced DLNN methods – ResNet101, EfficientNet and Vision Transformers (ViT) in coding CDT into binary and ordinal (0 to 5) scores. We extended beyond the traditional nominal classification approach (which does not recognize order) by introducing structured ordering into the coding system and compared DLNN-coded CDT images with manual coding. Results suggest that ViT outperforms ResNet101 and EfficientNet, as well as manual coding. The ordinal coding system has the ability to allow researchers to minimize either under- or over-estimation errors. Starting in 2022, our developed ViT-coding system has been used in NHATS’ annual CDT-coding.

## INTRODUCTION

Alzheimer’s disease and related dementias (ADRD) is a set of conditions, most often occurring in later life, that impair cognition and functioning. Nearly 6 million people in the U.S. and 50 million people worldwide are affected.^1,2^ With the population worldwide aging, the number of people living with ADRD is expected to increase dramatically in the coming years,^3^ posing substantial challenges to the families and health care systems that support them. Because there is no single test for ADRD, multiple approaches have been developed to screen for dementia. Since the 1990s, the clock-drawing test (CDT), which asks subjects to draw a clock (typically with hands showing ten after 11), has been recognized as a reliable tool to evaluate a wide range of cognitive functions either by itself or as part of a brief battery and has been used in clinical research, epidemiologic studies and panel surveys.^4^ The CDT has been favored because it requires little training for clinicians and researchers to implement, can be administered in less than two minutes and requires only paper and a writing utensil.^5^ Several scoring algorithms for the CDT exist, but generally the clock is assigned an overall ordinal score or count of ordinal subscores (points) for various elements.^6,7^

Nevertheless, barriers exist to taking fuller advantage of the CDT. For example, because the CDT requires manual coding, when used in large studies, biases may be introduced if there are systematic differences in how coders implement rules (i.e., “coder effects”). This issue is especially a concern in longitudinal studies in which clock drawings by the same individual may be coded inconsistently over time by different coders. In addition, some researchers argue that existing CDT scoring algorithms are generally not suitable for detecting mild cognitive impairment (MCI).^8,9^ Qualitative CDT coding (i.e. subjective evaluation of error sources) can better distinguish MCI but is time-consuming and subject to larger coder effects than standard coding.^10^

The rapid development of deep learning neural networks (DLNN) in the past several years and advancement of image coding^11^ in particular make this technique ripe for exploring application to CDT-coding with large-scale, representative samples, which traditionally relies mainly on manual coding.

The application of DLNN to CDT-coding has two potential advantages over manual-coding of CDT. First, DLNN has the potential to reduce resources needed to incorporate clock codes into large-scale studies. Manual coding currently requires recruitment of coders, training, evaluation, and certification, coding (in which the coders need to review, interpret and score each CDT), and quality checks. Given its critical role in dementia detection and the wide range of research areas to which CDT data have contributed,^5,12–19^ it would be advantageous if the resources needed to include the CDT in large-scale studies could be reduced. Second, DLNN has the potential to produce codes with higher reliability and validity than manual coding. Manual coding is subject to errors and inconsistencies because coder interpretations of CDT and the scoring system may be different or applied inconsistently. In longitudinal studies, errors in manual coding can be inconsistent over time. In contrast, studies have shown that well-established machine learning methods can classify images more reliably and more accurately than humans.^20–23^

Applications to automate CDT-coding have been mostly attempted with small and non-representative respondent pools but thus far have yielded inconsistent accuracy rates and have mainly focused on binary coding: normal vs. abnormal.^24–31^ While a scarce number of studies have explored the coding of CDT into ordinal categories and have some promising results, ^32–34^ these studies are limited in three important ways. First, despite the ordinal nature of CDT scores where class labels include information about the relative ordering between the labels, none of the previous studies have considered this ordinal feature; instead, these studies all used the standard nominal classification loss functions such as multi-category cross-entropy to code DLNN. Moreover, CDT coding studies attempting classification into more than two categories have yielded relatively low accuracy. Second, with very few exceptions, ^35–37^ most of these studies have been conducted using nonrepresentative and small-scale samples, with limited racial/ethnic diversity and narrow ranges of cognitive impairment. Third, many of these studies assume that manual-coded CDT scores are error-free, and do not acknowledge the possibility of coder effects.

### DLNN models for Enhanced CDT-Coding

This study investigated three advanced DLNN models to code CDT– ResNet101, EfficientNet and Vision Transformer (ViT) combined with transfer learning. ResNet101 and EfficientNet belong to the Convolutional Neural Network (CNN) family, the primary deep learning architecture in recent years for computer vision tasks. While other deep learning neural network (DLNN) models such as GoogLeNet and Inception-v3 aim to reduce computational cost by ‘going deeper’ to decrease parameters while maintaining performance, ResNet stands out for its ability to train extremely deep neural networks (comprising hundreds or even thousands of layers) without encountering issues like vanishing gradients or degradation. EfficientNet was designed to achieve better performance by scaling the network’s depth, width, and resolution simultaneously, resulting in models that are both efficient and effective across a wide range of tasks ^38,39^. Based on previous research, ResNet101 and EfficientNet outperformed other well-known NN models (e.g., VGG, GoogLeNet, Inception, MobileNets, Densenets and NASNet) in a number of computer vision tasks in terms of achieving better accuracy and increased computational efficiency. ^39,40^ Further details are provided in the Methods section.

### Integrating Vision Transformer in CDT-coding

Unlike ResNet101 and EfficientNet, ViT is a type of neural network architecture that uses a pure transformer applied directly to sequences of images patches for image classification tasks. Previous research^41–43^ demonstrated that the reliance on CNNs is not necessary, and a pure transformer applied directly to sequences of image patches can perform very well on image classification tasks. When pre-trained on large amounts of data and transferred to multiple mid-sized or small image recognition benchmarks (ImageNet, CIFAR-100, VTAB, etc.), ViT attained excellent results compared to the state-of-the-art convolutional networks while requiring substantially fewer computational resources to train. To the best of our knowledge, this is the first study applying ViT to CDT-coding.

### An innovative multi-class ordinal coding

The current practice of image classification through deep learning has predominantly relied on a nominal classification approach, assigning exclusive, non-ordering labels to images. However, this classification methodology overlooks the intrinsic ordering of categories in CDT scores. Consequently, this method, despite employing a more general model, may encounter challenges such as conflicting probabilities for various categories and the possibility of overfitting.

In this study, we introduce structured ordering into the coding system. The approach has the potential to minimize classification errors as it better mirrors the comparative thinking employed by human coders evaluating the CDT images. The ordinal coding approach also allows researchers to control the direction of errors (minimizing over- or under-estimation).

The ordinal CDT-coding belongs to a particular type of supervised learning problems called ranking or ordinal classification, which considers the inherent order of outcomes. Specifically, a nominal classification problem can be defined as building a system that maps an input space X to a class label space, $L=\left\{l_{1},l_{2},\ldots ,l_{k}\right\}$. Unlike traditional nominal classification, an ordinal classification system maps an input space X to an ordinal class label space, $OL=\left\{l_{1},l_{2},\ldots ,l_{k}|l_{1}<l_{2}<\ldots <l_{k}\right\}$. While the field of machine learning has developed many powerful algorithms for predictive modeling, most of these algorithms were designed for nominal classification tasks, where the commonly-used loss functions in these algorithms is multi-class cross-entropy, which do not capture the ordering properties contained in the label space.

Although no previous studies have applied ordinal DLNN approach to CDT-coding, a number of ML techniques have been developed to address ordinal classification problems. Li and Lin (2007) proposed a reduction framework from ordinal regression to binary classification based on extended examples.^44^ The extended binary classification approach forms the basis of many ordinal regression implementations. However, neural network-based implementations of this approach commonly suffer from classifier inconsistencies among the binary rankings.^45^ This ordering property cannot be captured by commonly used loss functions such as multi-category cross-entropy in DLNN classification systems in some cases, as shown in Cao et al. (2020).^46^ In this paper, we investigate innovative methods for placing constraints of classifier consistency that can easily be implemented in various deep learning neural network architectures, and new metrics for evaluating ordinal classification systems.

### Research questions

The nature of ordinal data in CDT scores prompts consideration between deploying a more general classification model with fewer constraints and a less general ordinal model with more constraints. Our research pioneers a comparative study of image classification and ordinal coding using DLNN for assigning CDT scores, providing insights into the potential applications of our developed ordinal coding for other image classification tasks with ordinal outcomes.

Using what is believed to be the world’s largest publicly available repository of CDT images, from the 2011–2019 National Health and Aging Trends Study (NHATS), this study attempts to address the following research questions:

1. Which DLNN models are most effective in coding CDT scores using 1) binary (impaired, non-impaired) scoring and 2) ordinal scoring with 6 classes? To address this question, we explored state-of-the-art DLNN technology for CDT classification and compared performances of ResNet101, EfficientNet and Vision Transformer.
2. Does ordinal DLNN coding outperform traditional nominal classification? To address this question, we modified DLNN models using an ordinal-coding approach and compared the standard nominal approach with the newly developed ordinal approach. We also performed a sensitivity analysis to demonstrate the ability of the ordinal approach to allow researchers to shift errors toward over or under estimation of cognitive function.
3. How do the DLNN coded CDT scores compare to manually coded scores? To address this question, we evaluate the performance of the ResNet101, EfficientNet and Vision Transformer techniques relative to human coders for both binary and ordinal scoring.

## DATA

### NHATS CDT data collection.

We used 9 rounds of data from NHATS, a nationally representative panel study of adults ages 65 and older living in the U.S. NHATS was initiated by the National Institute on Aging in 2008 to guide efforts to reduce disability, maximize health and independent functioning and enhance quality of life at older ages.^47,48^ The sample was drawn from the Medicare enrollment file, which covers approximately 96% of older adults in the U.S. Respondents were sampled for Round 1 (2011) and replenished in Round 5 (2015) and Round 12 (2022) using a stratified three-stage sample design in which individuals at older ages and Black individuals were oversampled.^49^ In total, 8,245 respondents participated in Round 1. The response rate was 71% in Round 1, 77% in Round 5, 59% in Round 12 and exceeded 85% in other rounds.^48^

NHATS has collected pen-and-paper-based CDT items annually, where respondents have two minutes to draw a clock showing 10 past 11. In total, more than 47,000 CDT images are available for Rounds 1 to 9. On average across these rounds, about 50% of clocks were drawn by respondents age 80 or older, 60% by females, and about 30% by non-White (21% Black) individuals. Based on the full cognitive battery,^50^ 11% were drawn by someone classified as having probable dementia and 11% as having possible dementia.

### CDT Coding.

Once collected, clocks were scanned into an online database for coding. Clocks were coded by trained lay coders on an ordinal scale as follows: 0 Not recognizable as a clock, 1 Severely distorted depiction, 2 Moderately distorted depiction, 3 Mildly distorted depiction, 4 Reasonably accurate depiction, and 5 Accurate depiction of a clock, using a coding system developed by Psychological Assessment Resources, Inc.^51^ Illustrations of clocks coded with various scores are included in Appendix Fig. 1. Each round, coders participated in a two-hour CDT training session and were asked to code 219 training clocks. The 219 clocks were also coded by two neuropsychology fellows, which were considered the gold standard given their clinical background in cognitive assessment. The Cohen’s weighted Kappa for the inter-coder reliability between each lay coder and the neuropsychology fellows was calculated and used to select qualified coders. The final number of coders selected for each round and the minimum Cohen’s weighted Kappa score to select coders for each round are included in Table 1.

### Data Description.

Two datasets were used in this study. The first dataset contains CDT data collected from NHATS respondents from Round 1 to Round 9. This dataset is used to train the DLNN models and evaluate the performances of these models. As shown in Table 1, in total, over 30,000 CDT images were collected in Rounds 1 to 9. To ensure the use of high-quality images and coded scores, we selected CDT images based on the following two criteria: 1) Images categorized as having good clarity by coders, and 2) CDT images coded by the eight top-ranked coders, all of whom have maintained an average Kappa score exceeding 0.75 across the years. In total, 25,872 CDT images were selected. Selected coders and their Kappa scores are presented in Appendix Table 1.

The second dataset includes the aforementioned 219 CDT images, which were used to evaluate the performances of DLNN models compared to NHATS coders. We refer this dataset as “benchmark data” in this paper. Notably, all coders coded the 219 CDT images in each year that they participated in coding, and two neuropsychology fellows also coded them. This dataset provides an opportunity to evaluate coder effects and compare DLNN models with human coders.

## METHODS

**Figure 1:**
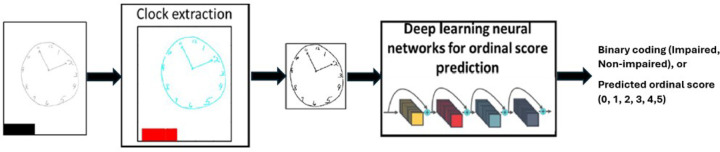
An automatic CDT scoring system based on CS_Net, a DLNN for ordinal score prediction. Note. All CDT images in this paper are drawn by the research team for illustration purposes. No individual respondent data were shown in this paper.

To automatically code CDT using DLNN, we developed an entropy-based DLNN system, which we call CS_Net (an overview is provided in Fig. 1). The CS_Net consists of two subsystems: Clock Extraction (ClE) system, and a multi-layered DLNN model trained for CDT score prediction. The Clock Extraction (ClE) system extracts the clock from the scanned image. The multi-layered DLNN model typically contains two processing blocks: 1) feature embedding and 2) classification. In deep neural networks, the feature embedding block contains multiple layers of neurons. It transforms input data, which could be of high dimensionality and/or have complex structures, into a lower-dimensional and more compact representation while preserving important information. The structure of the feature embedding block varies much depending on the nature of the data and the specific problem one is trying to solve. Feature embedding is crucial for many deep learning applications, as they help transform raw data into a format that the classification block can effectively learn to perform classification or prediction tasks. The output of the CS_Net system is the predicted CDT score or a binary classification code. The following subsections provide detailed descriptions of the computational components in CS_Net.

### Clock extraction

The original CDT images often contain various irrelevant marks, including masked IDs, lines on the drawing paper and incidental drawing. We developed an automatic clock image segmentation system that consists of two major components, image preprocessing and clock extraction. The image preprocessing component consists of mathematical morphology operations, erosion and dilations, to detect and remove the lines from the input image. It also includes a clustering process to group all the black pixels within a small distance into several connected components. The clock extraction process detects clock drawing based on the clock segment’s black pixel density and the ratio, r, of the segment’s width and height. Specifically, a component is considered containing the clock drawing if its density is less than 0.6 and its |r-1|<0.4.

### DLNN ordinal classification models for CDT score prediction

**Figure 2:**
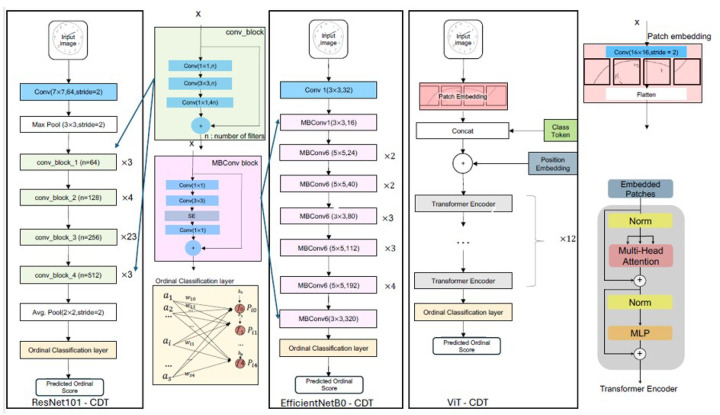
A unified DLNN framework for ordinal CDT classification

Figure 2 illustrates the DLNN based ordinal classification system, for automatically classifying clock drawings. We present three DLNN based ordinal classification systems: two CNN-based ordinal classification systems, Resnet101-CDT, EfficientNetb0-CDT, and an attention-based transformer system, ViT-CDT. The structure of Resnet101-CDT consists of multiple convolutional blocks, and its details are illustrated in the “conv_block.” EfficientNet is a family of CNN architectures. EfficientNetb0-CDT consists of multiple MBConv blocks, and its details are illustrated in the “MBConv block.” The ViT-CDT consists a patch encoding layer and multiple Transformer Encoders, and the details of these blocks are also illustrated in the figure. All three DLNN CDT systems take the clock drawing image as input, all pass it through their respective computational blocks and the final Ordinal Classification layer, which is also illustrated in the figure.

Under the CS_Net framework, each DLNN model incorporates constraints of ordinal consistency in its learning process. In addition, we also compared the aforementioned ordinal systems with the corresponding transformer-based and CNN-based nominal classification systems for two tasks: (1) screening dementia (binary classification of CDT images) and (2) scoring dementia into six categories.

### Machine learning in ordinal score classification

Machine learning for ordinal CDT score classification can be described as follows. Let the training data set containing N samples be $D=\left\{\left(x_{i},l_{i}\right)|i=1,\ldots ,N\right\}$, where $x_{i}$ is the extracted digital image of a clock drawing by respondent i, and $l_{i}$ is a coded category, $l_{i}=k\in \{0,\;1,\;2,\;3,\;4,\;5\}$, which corresponds to the six ordinal CDT scores. For an input image $x_{i}$, its coded category $l_{i}=k$ is extended to a rank ordered label vector, $\backslash varvecl_{\backslash varveci}=\left(yl_{i}^{0},yl_{i}^{1},\ldots ,yl_{i}^{4}\right)$, where $yl_{i}^{j}=1$ for j=0,…,k, and $yl_{i}^{j}=0$ for j>k, where 0≤j<5. A DLNN model for this application contains feature extraction layers and an output layer of five binary classifiers, each is trained to generate a correct prediction of the binary output for $yl_{i}^{k}\in \{0,\;1\},$ k=0,…,4. The prediction generated by the $k^{\text{th}}$ binary classifier is denoted as $P\left(yl_{i}^{k}=1\right)=\sigma \left(\sum_{s=0}^{4}a_{is}w_{s}+b_{k}\right)$ where $yl_{i}^{k}$ is the k-th binary classifier’s result generated from input feature vector $x_{i}$. If $\left[yl_{i}^{0},yl_{i}^{1},\ldots ,yl_{i}^{4},\right]=[1,0,\;0,0,\;0]$, it implies that $x_{i}$ has a rank of 1 and if $\left[yl_{i}^{0},yl_{i}^{1},\ldots ,yl_{i}^{4}\right]=[1,\;1,\;1,\;1,\;1]$, it implies that $x_{i}$ has a rank of 5. $a_{i1s}$ are the output of a DLNN model, i.e. EfficientNet, ResNet 101 or ViT, with respect to the input $x_{i},$ $w_{s}$ are the weights parameters in the DLNN, $b_{k}$ is the bias vector of the final layer, where the non-linear sigmoid function σ is used to generate a value from 0 to 1 range.

### Deep Learning Neural Network Models

ResNet, short for Residual Network, is a type of deep neural network architecture characterized by the use of residual blocks, which address the challenges of training very deep neural networks by introducing skip connections.^40^ The developed ResNet101-CDT is part of the ResNet family, it consists of 101 layers, including both convolutional and fully connected layers with the input being a CDT image. Its architecture consists of an initial convolutional layer that performs a convolution operation on the input image to extract basic features followed by batch normalization and a rectified linear unit (ReLU) activation function and multiple residual stages. Each residual stage contains several residual blocks. The number of residual blocks in each stage is determined by the architecture design. ResNet101-CDT is composed of multiple residual stages, each containing several residual blocks. The number of residual blocks in each stage is determined by the architecture design. The residual blocks within each stage have skip connections that allow the gradient to flow more easily during training. Each residual block consists of two or more convolutional layers with batch normalization and ReLU activation functions. The key innovation is the introduction of a skip (or shortcut) connection, allowing the output of one layer to bypass several layers and be added directly to the output of deeper layers. The skip connection helps in mitigating the vanishing gradient problem, making it easier to train very deep networks. Towards the end of the network, a global average pooling layer is applied to convert the spatial features into a vector by averaging them. This reduces the spatial dimensions and retains essential information. The final layer is a fully connected layer with 6 neurons to produce the output logits for the CDT class labels. In this research we trained the ResNet101-CDT using the cross-entropy loss and optimized using stochastic gradient descent (SGD) with momentum.

The EfficientNetB0-CDT we implemented for CDT classification is one of the eight EfficientNet models, EfficientNetB0, B1,..., B7, proposed by Tan et al. (2019).^39^ These EfficientNets were designed with different scaling strategies such that the depth, width and resolution of each network are uniformly adjusted for different tasks. The basic building block of EfficientNetB0-CDT, referred to as the Efficient Block, incorporates depth-wise separable convolutions, squeeze-and-excitation blocks, and linear projections to optimize model efficiency.^39^ The depth-wise separable convolutions reduce the number of parameters, and the squeeze-and-excitation blocks enhance feature recalibration. An EfficientNet typically consist of multiple blocks and stages, each contributing to the extraction of increasingly abstract features. As it progresses through the network, the receptive fields of neurons become larger, enabling the model to capture more global context and relationships between different parts of the image.^38^ Aa global average pooling layer is typically used towards the end of the network. This reduces spatial dimensions and retains essential information for classification. The final layer is a fully connected layer with 6 neurons to produce the output logits for the CDT class labels. The depth-wise separable convolutions reduce the number of parameters, and the squeeze-and-excitation blocks enhance feature recalibration.

**Figure 3:**
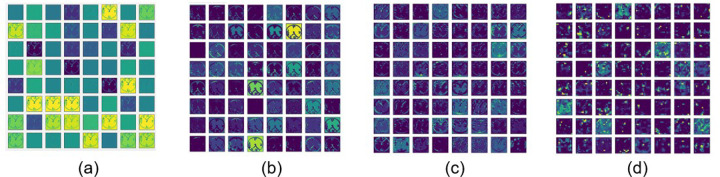
An example of clock drawing features generated by convolution layers in Resnet101: (a) Feature map generated by the first convolutional layer, (b) Feature map generated by the first Residual module (c) Feature map generated by the second Residual module, (d) Feature map generated by the third Residual module.

When CDT image samples are input to either Resnet101-CDT or EfficientNetB0-CDT, there are two major computational steps taking place for CDT classification. The first step is feature extraction. The feature extraction process identifies features that generally characterize each target class and discriminates the target class from the other classes. The second step is to conduct the classification task using the extracted features. In ResNet101-CDT and EfficientNetB0-CDT, the initial convolutional layers are used to capture low-level features such as edges and basic textures. These layers serve as the feature extraction backbone and are responsible for transforming the input image into a set of higher-level representations. An example of such features generated by various layers in Resnet101 from one CDT image is presented in Fig. 3. The extracted features are sent to the fully connected layers as input to generate the probabilities that the input image belongs to the corresponding classes. The classification layer has 6 nodes for the CDT score prediction, and 2 nodes for binary classification.

The ViT-CDT system we developed for CDT classification is modeled after the ViT architecture presented in Dosovitskiy et al (2020).^41^ Different from the ResNets and EfficientNets, ViT-CDT system does not contain any convolutional or recurrent layers. The major computational components include a feature embedding layer, a transformer encoder, and a classification layer. The input to the ViT system is sequences of 196 image patches, each is 16×16 in size, generated from a CDT image. The input embedding is concatenated with the positional encoding vector, and the result is a sequence of vectors that is used as input to a standard Transformer as illustrated in Fig. 2. The Transformer encoder is a stack of identical encoder blocks, each containing a multi-head self-attention (MSA) layer and a position-wise feed-forward neural network (FNN). Specifically, the encoder self-attention has the inputs taken from the outputs of the previous encoder block, except the first one, which takes the output from the embedding layer. A residual connection is applied to both MSA and FNN. Finally, a classification layer is connected to the last encoder block to generate predicted classes. The multi-head attention layer is trained to learn different patterns contained in the input sequence, such as capturing dependencies between the image patches at different positions.

We implemented the three DLNN models, ResNet101-CDT, EfficientNetB0-CDT and ViT-CDT in three types of CDT classification frameworks: binary, nominal and ordinal.

### Transfer Learning

All three DLNN models are trained using a transfer learning approach, which uses ImageNet as the source data in pre-training and then the CDT data at the fine-tuning stage. ImageNet is a large dataset containing over a million labeled images across 1,000 classes. The pretrained weights capture object features through diverse image classification tasks, which can be used to accelerate our model’s training procedure by leveraging the information distilled from ImageNet’s extensive dataset.

The fine-tuning stage attempts to learn hierarchical features through two phases. At phase 1, since the first layers contain the low-level features that are universal and generalize well over all classes, e.g. curves /edges / blobs, therefore these weights are kept intact, only the weights at the final classification layer are updated using the CDT training images for M epochs, where M is a hyperparameter.

At phase 2, weights in all layers are optimized using the same loss function based on the nuances and specifics of the CDT image classification. Different learning rates are used at different layers. Smaller learning rates are applied to the early convolutional layers and a larger learning rate is applied to the classification layer.

The loss function used in fine tuning the nominal classifiers is multi categorical cross entropy function:

$$L_{n}=-\frac{1}{N}\sum_{i=1}^{N}\sum_{k=0}^{5}y_{i,k}*log{\;\overset{ˆ}{y}}_{i,k}$$

where N is the number of samples in a mini batch, $y_{i,k}$ is an binary indicator of the true status of the input CDT image $x_{i}$ being class k. And ${\overset{ˆ}{y}}_{i,k}$ is the predicted probability of the input image $x_{i}$ belonging to class k. The loss function is used in fine tuning the nominal classifiers.

The loss function used in ordinal coding system is

$$L_{o}=-\frac{1}{N}\sum_{i=1}^{N}\sum_{k=0}^{4}(1-\alpha )y_{i}^{k}*log{\overset{ˆ}{y}}_{i}^{k}+\alpha \left(1-y_{i}^{k}\right)*log(1-{\overset{ˆ}{y}}_{i}^{k})$$

where $y_{i}^{k}$ is a binary indicator for the true status of the input CDT image $x_{i}$ has $rank>k,\;{\overset{ˆ}{y}}_{i}^{k}=\sigma \left(g\left(x_{i},W\right)+b\right)$ is the predicted probability of the input image $x_{i}$ having rank>k, and N is the mini batch size, which can vary at the two learning phases.

Note that varying the α parameter in the loss function for ordinal coding system allows for a deliberate trade-off between errors in different directions – overestimation versus underestimation of cognitive functions. In this study, we also conducted a sensitivity analysis to examine the influence of α parameter on the performance of the DLNN models as captured by the overestimation error rate.

### Performance metrics

#### DLNN model comparisons.

For binary CDT coding, we compared the aforementioned DLNN models using five metrics: accuracy, sensitivity, specificity, positive likelihood ratio, and negative likelihood ratio. Higher values in each of these metrics indicate better performances.

For six-category CDT-coding, we used four performance metrics: accuracy, root mean square error (RMSE), gamma coefficient (γ), and weighted Kappa. Specifically, $RMSE=\sqrt{\frac{\sum (\mathrm{pred}\;-\mathrm{ground}\;\mathrm{truth}{)}^{2}}{\mathrm{total}\;\#\;\mathrm{of}\;\mathrm{samples}}}$ which measures the average absolute deviation between the real and predicted score; γ measures the association between two ordered categorical variables, which is calculated as $\gamma =\frac{C-D}{C+D}$, where C is the number of concordant pairs in predictions, and D is the discordant pairs in predictions; and weighted Kappa measures the agreement between two coding methods. For accuracy, we examined accuracy of correct prediction of the score, and two additional accuracy measures: 1) $Acc_{r+1}$, the accuracy above the diagonal by 1, is calculated as the ratio of the number of images that are correctly predicted or predicted as r+1, where r is the ground truth class score of the input image, to the total number of images in the test data; 2) $Acc_{r\pm \;1}$, the accuracy off diagonal by 1 is calculated as the ratio of the number of the images being correctly predicted or predicted as r±1, where r is the ground truth of the input image with the total number of images in test data. This measure allows the estimated score to be within a neighboring class of ± 1 of the true label. Higher accuracy, γ and weighted Kappa, and lower RMSE indicates better performance.

For the sensitivity analysis on the change on the direction of errors (penalizing underestimation vs. overestimation), we examined the overestimation error rate, which measures the proportion of CDTs predicated higher than the manual codes.

#### Comparisons with manual coding.

Using expert coding as the benchmark, we compared the three DLNN models against NHATS manual coding for both binary and six-category CDT scoring. For binary CDT coding, accuracy for DLNN models and manual coding were examined. For six-category CDT coding, weighted kappa scores were used. A total of 15 NHATS coders coded the 219 benchmark CDT images at least once across Rounds 1 to 9. The manual coding results were calculated by averaging the accuracy or kappa measures across all coder-year evaluations (see also Appendix Table 1).

## RESULTS

### Image Segmentation Result

A total of 25,872 images were extracted across Rounds 1 to 9, with about 95% of them automatically processed by our developed clock image segmentation system. About 5% of these images cannot be successfully extracted and were manually extracted, mainly due to unconnected circle lines and multiple clock drawings.

### Experiments on automatic CDC-coding

We present the results generated by applying DLNN models to two types of CDT-coding: 1) binary classification, and 2) six-category scoring. For the latter, we compared our developed ordinal coding approach with the traditional nominal classification approach using DLNN. The binary coding is used to differentiate between non-impaired vs. impaired results, where scores of 0 to 3 are considered as impaired and scores of 4 or 5 as non-impaired. Six-category scoring involves assigning a score of 0 to 5 for each CDT, using ordinal or nominal classification-based DLNN. We combined images in classes 0, 1, 2 and 3 into the impaired group, and images in classes 4 and 5 into the non-impaired group.

The 25,872 segmented clock images were partitioned into a training set and a test set using stratified random sampling method with the 90% vs.10% split. The distribution of clock image segments in the training and test sets are shown in Table 2.

All six DLNN models were built through transfer learning on the training data. In the implementation, we used pretrained weights on ImageNet Dataset released by reputable computer vision frameworks PyTorch and TensorFlow. The hyperparameters used in the transfer learning processes are listed in Appendix Table 2.

In the following evaluation, we compared the performances of different neural network architectures and models with the training and test data described above.

### CDT binary coding results

Binary classification systems were trained as follows. There are 14,582 CDTs classified as non-impaired and 7,909 CDTs as impaired in the training set. The corresponding numbers for the test data are 1,620 for non-impaired CDT and 880 for impaired drawings. The performances of the four binary classifiers were trained on the same training data set and evaluated on the same test data, and the results are shown in Table 3.

All three models – ResNet101-CDT, EfficientNetB0-CDT, and ViT-CDT – achieved good accuracy measures, where ResNet101 and ViT have the highest accuracy at 98.4%, closely followed by EfficientNetB0-CDT at 97.8%. ViT and ResNet101 have the highest accuracy and sensitivity, comparing to EfficientNetB0.

In comparison to the performance of DenseNet-121, which was the best system presented in Chen et al. (2020)^33^ and achieved 96.65% classification accuracy over a small test data of 263 CDT images. Our experiments showed that both the ResNet101 and ViT-ordinal gave 1.8% better than the DenseNet-121 over 2,500 CDT images.

### CDT ordinal coding results

Table 4 presents the performance of ResNet101, EfficientNet, and ViT models in coding the six-point CDT score using both nominal classification and ordinal coding approaches. Among the three models, ViT outperforms both ResNet101 and EfficientNet in all performance metrics, with the highest accuracy (77.5%), lowest RMSE (0.54), highest gamma (0.94), and highest weighted kappa (0.79). For ResNet101 and EfficientNet, ordinal approach outperforms classification approach with lower RMSE, higher accuracy, gamma coefficients and kappa. For ViT, both classification and ordinal approaches achieved similar performances.

For ResNet101 and EfficientNet, $Acc_{r+1}$ has improved accuracy more than 15%, and ViT has improved more than 12%. The ordinal operators give better predictions than their counterpart in each DLNN model. This accuracy measure $Acc_{r\pm 1}$ is very high for all classifiers. The ViT ordinal classifiers reached 98% in $Acc_{r\pm 1}$ over 2,500 test images, which is comparable with the results presented in Chen et al. (2020),^33^ which reported a 98.54% off diagonal accuracy over about 250 test images.

**Figure 4:**
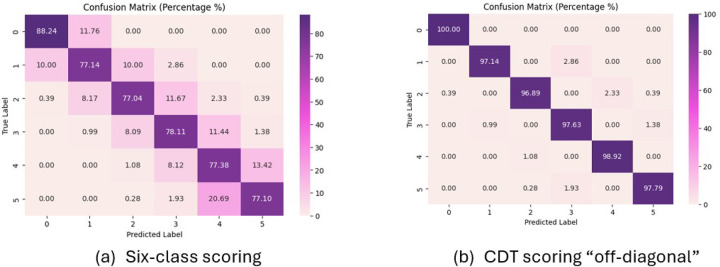
Confusion matrices generated by ViT ordinal classification on the test set

Figure 4 presents the confusion matrices generated by the ViT ordinal coding system, where (a) is the confusion matrix for scoring CDT in six classes, and (b) is the confusion matrix that considers the off-diagonal by 1 point as correct. Results show that the model has a good performance, with overall accuracy for a) as 77.5% and b) as 98.1%.

### Sensitivity analysis

Table 5 shows the accuracy, RMSE, overestimation error rate, accuracy for r+1, and accuracy for r±1 for both ViT nominal and ViT ordinal with different α parameter values, which can shift errors in different directions. Overestimation error measures the chances that a classification system predicts CDT scores that are higher than the images’ true score, i.e., predicts less impaired than true level of impairment. With α ranging from 0.5 to 0.75, the overestimation error rate decreases. When the α coefficient value is at 0.5, both under- and over-estimation error rates are equally penalized. As the α coefficient increases, the overestimation error rate is penalized more than the underestimation error. This method is very useful when researchers would like to minimize overestimation error and identify more individuals with impairment for further screening.

The optimal result of accuracy and RMSE of ordinal and nominal models are similar as accuracy = 77.5% and RMSE = 0.55, which is achieved at α = 0.55. When the α value keeps increasing, there is a slight decrease of the accuracy metric. The RMSE value remains consistent for different α values, only with a slight increase to 0.57 at α = 0.75.

When comparing the accuracy above diagonal by + 1 and ± 1 class of nominal ViT, all the ordinal models except when α = 0.50 are more conservative and less likely to overestimate CDT score than nominal ViT. Within the ordinal ViT performances, the overestimation error rate consistently decreases from 0.15 to 0.09, indicating that the model is making fewer errors in generating false positive CDT scores.

### Comparisons with manual coding

Table 6 compares CDT binary coding accuracy of the three Deep Learning Neural Network (DLNN) methods against average manual coding results of 15 NHATS coders who coded the benchmark clocks in one or more years (see Appendix Table 1). With two experts’ coding as the gold standards, ViT and EfficientNet achieved slightly lower but similar accuracy compared to manual coding, while ResNet101 achieved the lowest accuracy (95.7%).

Table 7 compares performances of the three Deep Learning Neural Network (DLNN) methods against manual coding in coding the six-point ordinal scores. With two experts’ coding as the benchmark, ResNet101 and EfficientNet have lower weighted kappa scores with expert coding, comparing to manual coding. Only ViT outperformed most human coders in coding ordinal CDT with highest weighted Kappa scores, suggesting that ViT coded ordinal CDT most similar as expert coders. Note that the weighted kappa between the two expert coders is 0.81.

## DISCUSSION

The clock-drawing test (CDT) has been widely used as a screening tool to detect dementia in clinical research, epidemiologic studies, and panel surveys. An important limitation in the use of CDT in large-scale studies is that the CDT requires manual coding, which could result in biases if coders interpret and implement coding rules in different ways. This study developed an intelligent CDT Clock Scoring system that automatically code CDT images, and compared three advanced DLNN models - ResNet101, EfficientNet, and ViT - in coding CDT into both binary and ordinal scores. For binary CDT coding, all three models coded CDT with high accuracy and achieved similar coding accuracy as manual coding. For CDT coding, ViT outperforms ResNet101 and EfficientNet with the highest accuracy. Compared to non-clinically trained human coders, ViT also coded CDT more consistently with clinically trained expert coders. Results of this study suggest that DLNN methods have the potential to replace manual-coding of CDT in large-scale studies, and ViT has the potential to improve coding accuracy and consistency compared to manual coding. In addition, for ResNET101 and EfficientNet, ordinal DLNN coding outperforms the traditional nominal classification approach. For ViT, although nominal and ordinal DLNN models provided similar results, the ordinal ViT has the additional advantage of allowing researchers to deliberately shift the direction of predication error as desired, e.g., penalizing underestimation error over overestimation.

Our work is methodologically innovative in several ways. First, the systems were trained and evaluated on a larger CDT dataset than other ML systems, and performances exceeded the previous CDT six-category classification systems in terms of classification accuracies and order consistencies.^33^ Second, to the best of our knowledge, our work is the first study to develop an ordinal coding DLNN system for CDT and compare ordinal-coding with the traditional nominal approach. The promising results of ordinal DLNN system shed light on future studies predicting ordinal outcomes using DLNN. Third, we are also the first to investigate the use of the ViT method in CDT-coding. This novel application outperforms other DLNN models and has been successfully used in NHATS for its annual CDT-coding. ^52^ Fourth, using the ordinal coding approach allows us to flexibly adjust the direction of errors, underestimation vs. overestimation, in CDT-coding. This can be useful in many real-life dementia detection situations. If researchers would like to use CDT as a screening test for dementia, which aims to identify as many as people with impairment as possible for further testing of cognitive functions, then they could use a relatively larger α in the loss function to minimize overestimation rate.

This study is not without limitations. Despite the fact that we selected CDT images coded by top human coders to reduce coding errors in the training data, like all other CDT-coding studies, coder effects cannot be ruled out, mainly due to the subjectiveness of CDT-coding. That being said, the training data is not error free, and DLNN can learn these errors from manual coding. More work is needed to detect these errors and further improve coding accuracy. Our ongoing effort in this area includes using similarity-based metric learning to detect coding errors. ^53^

This study has significant practical implications. The successful development of DLNN models for CDT scoring suggests a promising avenue for automating the coding process, reducing potential coding errors introduced by human coders. Among the DLNN models, ViT stands out as a particularly effective choice for improving coding accuracy and consistency compared to manual coding. In fact, based on results of this study, NHATS Round 12 CDT-coding is largely completed by our developed CDT-coding system using ViT. ^52^ In addition, the DLNN programs developed in this study may offer a model for automating coding of other widely available drawing tests used to evaluate a variety of cognitive functions.

## Figures and Tables

**Table 1:** NHATS Clock Repository by Data Collection Year

Year	2011	2012	2013	2014	2015	2016	2017	2018	2019
Final # of coders*	3	4	3	4	5	4	5	5	4
Minimum Cohen’s weighted Kappa scores for coders	0.73	0.69	0.75	0.75	0.75	0.76	0.72	0.72	0.73
Number of clocks available	6,918	5,504	4,459	3,671	7,076	5,997	5,256	4,658	4,186

* Some coders coded clocks at multiple rounds. In total, there are 15 unique coders across 9 rounds.

**Table 2:** Distribution of clock image classes in training and test sets

Training set			Test set		
	Category Label	Count		Category Label	Count
Impaired: 7,909	0	203	Impaired: 880	0	23
	1	738		1	82
	2	2,319		2	258
	3	4,649		3	517
Non-impaired: 14,582	4	8,360	Non-impaired: 1,620	4	929
	5	6,222		5	691
	Total	22,491		Total	2,500

**Table 3:** CDT binary coding results for ResNet101, EfficientNet, and ViT

	ResNet101-CDT	EfficientNetB0-CDT	ViT-CDT
Accuracy	98.4%	97.8%	98.4%
Sensitivity	99.4%	99.0%	99.4%
Specificity	76.2%	71.4%	75.2%
Positive likelihood ratio	4.17	3.47	4.01
Negative likelihood ratio	0.008	0.014	0.008

**Table 4:** CDT ordinal coding results for ResNet101, EfficientNet, and ViT using both nominal classification and ordinal coding approaches.

	ResNet101		EfficientNet		ViT	
	Nominal	Ordinal	Nominal	Ordinal	Nominal	Ordinal
Accuracy	72.8%	74.2%	65.6%	72.0%	77.5%	77.5%
Acc_r+1_	89.3%	87.3%	83.4%	86.9%	88.9%	89.2%
*Acc*_*r*±1_	96.4%	97.2%	94.1%	98.1%	97.8%	98.1%
RMSE	0.63	0.60	0.76	0.59	0.55	0.54
Gamma	0.91	0.92	0.84	0.93	0.94	0.94
Kappa	0.74	0.76	0.65	0.75	0.79	0.79

**Table 5:** Sensitivity analysis with a parameter changes in ViT ordinal CDT-coding, comparing to ViT nominal approach.

	α_value	Accuracy	RMSE	Overestimation of CDT score	Acc (r + 1)	Acc (r±1)
ViT Nominal	n/a	77.5%	0.55	0.13	0.89	0.98
ViT Ordinal	0.50	76.7%	0.55	0.15	0.90	0.98
	0.55	77.5%	0.54	0.13	0.89	0.98
	0.60	76.5%	0.55	0.12	0.88	0.98
	0.65	76.5%	0.54	0.11	0.87	0.98
	0.70	75.4%	0.55	0.10	0.85	0.98
	0.75	74.6%	0.57	0.09	0.82	0.98

**Table 6:** Comparisons of CDT binary coding between manual coding and the three DLNN methods.

	Accuracy with Expert 1	Accuracy with Expert 2	Average
Manual coding	97.9%	98.2%	98.1%
ViT	97.3%	96.8%	97.0%
ResNet101	95.4%	95.9%	95.7%
EfficientNet	96.8%	97.3%	97.0%

Note. Manual coding results are averages across all coders who participated in NHATS CDT coding in Rounds 1 to 9.

**Table 7:** CDT ordinal coding results for ResNet101, EfficientNet, ViT using both nominal classification and ordinal coding approaches vs. human coders.

	Manual coding	ResNet101		EfficientNet		ViT	
		Nominal	Ordinal	Nominal	Ordinal	Nominal	Ordinal
Wgted Kappa with Expert 1	0.76	0.68	0.74	0.58	0.71	0.81	0.82
Wgted Kappa with Expert 2	0.75	0.69	0.73	0.54	0.71	0.82	0.81
Average	0.76	0.68	0.73	0.56	0.71	0.81	0.81

Note. Manual coding results are averages across all coders who participated in NHATS CDT coding in Rounds 1 to 9. Note that the weighted kappa between the two expert coders is 0.81.

## Data Availability

All data used in this study are available on NHATS website (www.nhats.org).
